# Morphology of male world cup and elite bouldering athletes

**DOI:** 10.3389/fspor.2025.1588414

**Published:** 2025-06-11

**Authors:** Pawel Draga, Paulina Trybek, Paulina Baran, Dominik Pandurevic, Alexander Sutor, Gudmund Grønhaug

**Affiliations:** ^1^ Institute of Measurement and Sensor Technology, UMIT-Private University for Health Sciences, Medical Informatics and Technology GmbH, Hall in Tirol, Austria; ^2^ Institute of Physics, University of Silesia in Katowice, Chorzów, Poland; ^3^ W.I.R. gemeinnützige GmbH, Hall in Tirol, Austria; ^4^ Department of Sport Food and Natural Sciences, Western Norway University of Applied Sciences, Sogndal, Norway

**Keywords:** bouldering, competitive climbing, body composition, anthropometry, somatotype, heath-carter method, ISAK protocol, LASSO

## Abstract

**Aims:**

To compare the somatic characteristics and somatotypes of elite bouldering athletes competing in World Cups and World Championships with national-level climbers and general adult population norms, and to identify anthropometric characteristics that differentiate performance levels in competitive climbing and distinguish climbers from the general adult population.

**Materials and methods:**

Anthropometric data were measured according to the ISAK protocol and somatotype was determined using the Heath-Carter method. Tissue composition and body proportions were examined using measurements of skinfolds, circumferences, widths and indices such as Ape Index and Arm Index. Thirty-four men participated in the study: 9 IFSC-ranked international level athletes and 25 national athletes. Statistical analysis used the Shapiro-Wilk test to assess the normality of the distribution, the Student’s t-test or Mann-Whitney U test to compare groups, LASSO regression to identify significant characteristics and Spearman’s correlation coefficient to examine correlations between variables.

**Results:**

International climbers demonstrated a significantly lower body fat percentage (14.4±2.00%) compared to national athletes (17.56±2.16%) and the general adult population (18.4±2.9%). Thinner skinfolds and smaller thigh and arm girths were found among the higher level climbers. Body proportions were more favorable in international athletes, who showed higher Ape (1.06 ± 0.02) and Arm Index values (46.22 ± 1.26) compared to national-level competitors (1.03 ± 0.03 and 44.98 ± 1.45, respectively). No significant differences were observed in somatotype profiles.

**Conclusions:**

International climbers differ from national athletes by having higher muscle mass, lower body fat, smaller limb girths, and shorter stature. The benefits of these characteristics and the influence of selection processes remain unclear. Notably, the low body fat in elite climbers likely reflects training adaptations rather than calorie restriction.

## 1 Introduction

The growing popularity of climbing, as well as its inclusion in the group of Olympic sports (1), has increased the interest of researchers in this discipline. Scientists have repeatedly attempted to explain the influence of morphological and motor skills on climbers’ performance. Sport climbing is a discipline that requires aspects of both endurance and muscular strength (2), combined with complex biomechanics of movement (4) consisting of multiple phases (3), as well as intricate techniques and tactics. The development of climbers’ performance also depends on mental abilities (5), along with energy-based motor skills (6). According to research, success in climbing depends on a number of factors, including finger and arm muscle strength (7), resistance to fatigue during isometric contraction (8), specific hip mobility (9), and body composition in which the importance of components such as low body mass, low levels of fat tissue, and an average body height, are emphasized (10). The influence of body composition on athletic performance is an element often pointed out by researchers as a significant determinant of athletic performance (11). In addition to body composition, body size has a major role in sport, which can differ significantly in training and non-training groups, as well as the proportions of its various components. In addition to the aforementioned elements of the body structure, researchers define the somatotype of athletes and compare it between top athletes and untrained groups (12).

Somatotype is defined as a method of quantitative description of body shape and composition (13). Currently, the most widely used somatotyping method is the Heath-Carter method (14). The role of somatotype in the performance of sport climbers has also been the subject of research (15). However, in the field of sport climbing there is a lack of comparative studies of top World Cup athletes with national level athletes and non-athletes in all climbing disciplines. The three climbing disciplines differ in terms of duration, technique, tactics, and energy systems used during the effort (16). The different requirements of the three climbing disciplines can also be reflected in significant disparities in the structure, composition, and body proportions of the athletes (17). These differences are particularly evident among high-elite climbers. High-elite lead climbers typically present the lowest body mass (63.3±8.4kg), body height (173.3±6.3cm), and BMI (21.0±1.9), with body fat levels averaging (7.97±4.83%), as reported by Ginszt et al. and Ozimek et al. (18, 19). According to Ginszt et al. and Michailov et al., high-elite bouldering athletes are slightly heavier (66.8±8.8kg), taller (176.1±7.6cm), and show a marginally higher BMI (21.5±2.2), with body fat percentages of (12.1±3.6%) (18, 20). Speed climbers report the highest values for body mass (70.3±6.18kg), height (177.63±6.42cm), and BMI (22.3±1.9), with body fat levels of (7.36±1.9%), as observed by Krawczyk et al. (21). An extended analysis of body composition and somatotype among national-level bouldering athletes, including comparisons to non-athlete adults, was conducted by Ozimek et al. using the Heath-Carter method (22). However, the available literature on somatotype characteristics in bouldering remains limited.

## 2 Materials and methods

The aim of the study was to identify somatic characteristics that most significantly differentiate athletes based on their competitive level. The study included 9 bouldering athletes who were IFSC World Cup semifinalists and 25 National Cup semifinalists. The national group consisted of Polish climbers, while the international group included athletes from Japan (4), Poland (2), Austria (2), and France (1). Participants were recruited via email, provided with detailed study information, and gave voluntary consent. All data were anonymized. Anthropometric measurements were conducted during competitions at different times of the day, taking into account the pre-competition context. Anthropometric measurements were conducted according to standardized protocols and are detailed in Table 1, with somatotype assessment using the Heath-Carter method (23). Anthropometric measurements were taken with the following equipment: body height with a SiberHegner anthropometer, skinfold thickness with Harpenden calipers, circumferences with a steel tape, wrist and bicondylar diameters with a small SiberHegner caliper, and body weight with a Tanita TBF 583 scale.

**Table 1:** Anthropometric variables measured in the study. X, ∑ of TS, SbS, SpS skinfolds × 170.18 ÷ BH; AG, CG, corrected girths (arm, calf); HWR, height-to-weight ratio; a, acromion; B, basis; v, vertex; tr, trochanter major; sst, suprasternale; sy, symphysion; $da_{3}$, third finger dactylion; BMI, body mass index; FM, fat mass; LBM, lean body mass. Formulas based on standard references: (22, 23, 32–36).

Category	Variable	Formula/measurement
Lengths & indices	Body height [BH] (cm)	Vertex to floor
	Arm length [AL] (cm)	Acromiale to dactylion
	Leg length [LL] (cm)	Trochanterion to floor
	Arm span [AS] (cm)	Dactylion to dactylion
	Torso length [TL] (cm)	Suprasternale to symphysion
	Arm index [ArI]	$\frac{a-da_{3}}{\left(B-v\right)}\times 100$
	Ape index [ApI]	$\frac{da_{3}-da_{3}}{\left(B-v\right)}$
	Leg index [LI]	$\frac{\left(B-tr\right)}{\left(B-v\right)}\times 100$
	Torso index [TI]	$\frac{sst-sy}{\left(B-v\right)}\times 100$
	Intermembral index [II]	$\frac{a-da_{3}}{\left(B-sy\right)}\times 100$
Breadths	Shoulder [SB] (cm)	Acromiale to acromiale
	Pelvic [PB] (cm)	Iliocristale to iliocristale
	Humerus [HB] (cm)	Epicondylion laterale to epicondylion mediale
	Femur [FB] (cm)	Epicondylion laterale to epicondylion mediale
Girths	Forearm [FG] (cm)	Midpoint between wrist and elbow
	Arm tensed [ATG] (cm)	Maximal circumference during contraction
	Arm relaxed [ARG] (cm)	Midpoint of relaxed arm
	Waist [WG] (cm)	Narrowest part of torso
	Thigh [TG] (cm)	Midpoint between inguinal crease and patella
	Calf [CG] (cm)	Maximal calf circumference
	Neck [NG] (cm)	Below laryngeal prominence
	Chest Inh. [CIG] (cm)	Maximal chest expansion
	Chest Exh. [CEG] (cm)	At end of normal expiration
Skinfolds &	∑ of 7 Skinfolds (mm)	TS, BS, SbS, AS, CS, PS, TS
body composition	Triceps [TS] (mm)	Vertical fold, midline posterior upper arm
	Biceps [BS] (mm)	Vertical fold, midline anterior upper arm
	Pectoral [PS] (mm)	Diagonal fold, mid-chest
	Subscapular [SbS] (mm)	Diagonal fold, below inferior angle of scapula
	S.iliac [SiS] (mm)	Diagonal fold, above iliac crest
	Abdominal [AS] (mm)	Vertical fold, 2 cm from umbilicus
	Calf [CS] (mm)	Vertical fold, medial calf
	S.spinale [SpS] (mm)	Diagonal fold, above anterior superior iliac spine
	Thigh [TS] (mm)	Vertical fold, midline anterior thigh
	Body weight [BW] (kg)	Measured with scale
	BMI	$\frac{\text{BW}}{(B-v{)}^{2}}$
	Density [D] (${g/\mathrm{cm}}^{3}$)	1.1567−[0.0717log⁡(BS+TS+%SbS+SiS)]
	FM (%)	$100\times \left(\frac{4.201}{D}-3.813\right)$
	FM (kg)	Derived from FM%
	LBM (%)	$100\times \left(1-\frac{\text{FM\textbackslash{}\%}}{100}\right)$
	LBM (kg)	BW-FM
	Rohrer’s index [RI]	$\frac{\text{BW}}{(B-v{)}^{3}}$
Somatotype	Endomorphy [Endo]	$-0.7182+0.1451X-0.00068X^{2}+0.0000014X^{3}$
	Mesomorphy [Meso]	0.858⋅HB+0.601⋅FB+0.188⋅AG+0.161⋅CG−0.131⋅BH+4.5
	Ectomorphy [Ecto]	0.732⋅HWR−28.58ifHWR≥40.75

Data were collected by the same trained researcher, who completed a one-year internship in anthropometry and physical profiling at the National Research Institute (Institute of Sport). The internship included practical training in standardized measurement protocols aligned with ISAK guidelines (24). To assess measurement repeatability and confirm high intra-evaluator reliability, the technical coefficient of variation (TCV %) was calculated based on three non-consecutive measurements at each site. According to ISAK Level 1 standards, this value was not allowed to be higher than the maximum acceptable limits—1% for girths and 7.5% for skinfolds (25, 26). Values exceeding these limits were considered invalid and excluded from further analysis. Mean TCV values for girths and skinfolds are presented in Table 2.

**Table 2:** Descriptive statistics of somatic, demographic, and climbing variables (Mean ±std, Min–Max, change %, p-values, TCV %; significant in bold) for World Cup (Int.) and elite national (Nat.) athletes.

Variable	Mean ± SD Nat. (*n*=25)	Mean ± SD Int. (*n*=9)	Min–Max Nat.	Min–Max Int.	Change %	p-value	TCV %
Body height [BH] (cm)	178.22 ± 5.89	172.40 ± 6.53	169–192.4	161.3–180.4	3.38	**0.019**	–
Arm length [AL] (cm)	80.06 ± 4.45	79.11 ± 4.45	73–92.5	72.4–86.8	0.36	0.917	–
Leg length [LL] (cm)	84.69 ± 4.45	82.33 ± 3.88	78.2–93.8	77.2–88.6	2.79	0.712	–
Arm span [AS] (cm)	184.28 ± 7.58	182.49 ± 8.97	170.2–197.5	166.2–193.6	0.98	0.567	–
Torso length [TL] (cm)	51.79 ± 2.45	50.70 ± 2.86	47.3–56	46.3–55.3	2.15	0.280	–
Arm index [Arl]	44.93 ± 2.07	46.22 ± 1.26	42.3–52.4	44.8–48.8	2.87	0.016	–
Leg index [LI]	52.24 ± 1.33	52.11 ± 1.25	49–55.13	50.5–54.2	0.25	0.792	–
Torso index [TI]	29.06 ± 1.04	29.41 ± 1.23	27.2–31.3	27.4–30.9	1.2	0.424	–
Intermembral index [II]	86.01 ± 3.66	88.72 ± 2.46	79.7–95.2	84.4–91.5	3.15	**0.049**	–
APE index [ApI]	1.03 ± 0.02	1.06 ± 0.02	0.98–1.08	1.03–1.08	2.91	**0.004**	–
Shoulder [SB] (cm)	40.82 ± 1.76	40.86 ± 1.33	36–43.9	38.5–43	0.10	0.952	–
Pelvic [PB] (cm)	27.74 ± 1.73	27 ± 3.26	25–31	23–34.5	2.67	0.394	–
Humerus [HB] (cm)	6.99 ± 0.37	6.97 ± 0.41	6–7.8	6.2–7.3	0.29	0.864	–
Femur [FB] (cm)	9.51 ± 0.47	9.34 ± 0.56	8.5–10.2	8.4–10.5	1.82	0.389	–
Forearm [FG] (cm)	29.82 ± 1.62	28.11 ± 1.47	27.3–32.7	26–31	6.1	**0.009**	0.76
Arm tensed [ATG] (cm)	34.55 ± 1.88	32.72 ± 2.20	30.7–37.7	29.5–36	5.6	**0.023**	0.85
Arm relaxed [ARG] (cm)	31.00 ± 1.80	29.17 ± 1.71	27.8–34.7	26.5–31.5	6.3	**0.013**	0.79
Waist [WG] (cm)	74.58 ± 5.99	74.56 ± 4.74	55–86.5	66–81	0.03	0.993	0.90
Thigh [TG] (cm)	53.47 ± 2.03	49.34 ± 2.87	49.2–57.1	44.2–52	8.34	**0.001**	0.83
Calf [CG] (cm)	36.25 ± 1.75	34.11 ± 0.89	33.6–40	33–35	6.3	**0.001**	0.76
Neck [NG] (cm)	37.14 ± 1.56	36.11 ± 2.33	34.4–40.2	31.5–40.5	2.77	0.149	0.80
Chest Inh. [CIG] (cm)	96.87 ± 5.99	89.78 ± 4.67	87.5–112.4	83.5–98.5	7.82	**0.003**	0.91
Chest Exh. [CEG] (cm)	89.29 ± 5.99	82.48 ± 3.68	79–104.2	77.5–88.8	7.62	**0.003**	0.88
∑ of 7 skinfolds (mm)	54.5 ± 9.2	41.7 ± 2.9	35.4–75.2	38.3–48.3	23.5	**0.001**	–
Triceps [TS] (mm)	5.47 ± 1.5	3.96 ± 0.9	3.1–8.7	2.6–5.5	27.6	**0.006**	4.92
Biceps [BS] (mm)	3.35 ± 6.7	2.51 ± 0.4	1.9–5.1	1.9–3.0	25.1	**0.001**	4.04
Pectoral [PS] (mm)	6.54 ± 1.6	4.77 ± 0.7	3.7–11.5	4.0–6.3	27.1	**0.002**	4.49
S.scapular [SbS] (mm)	8.30 ± 1.3	5.90 ± 0.7	6.0–11.1	5.0–6.8	28.9	**0.001**	5.02
S.iliac [SiS] (mm)	6.60 ± 1.8	4.81 ± 0.5	3.5–10.6	3.8–5.5	27.1	**0.001**	6.07
Abdominal [AS] (mm)	7.76 ± 2.3	5.40 ± 0.6	4.8–12.5	4.5–6.2	30.4	**0.001**	5.54
Calf [CS] (mm)	5.23 ± 1.3	4.84 ± 0.9	3.3–9.0	3.8–6.0	7.46	0.402	4.88
S.spinale [SpS] (mm)	4.41 ± 0.8	3.46 ± 0.8	2.6–6.2	2.4–4.7	21.5	**0.006**	5.49
Thigh [TS] (mm)	6.84 ± 1.8	6.01 ± 1.1	3.7–9.8	4.9–8.5	21.5	0.219	6.32
Body weight [BW] (kg)	69.18 ± 6.11	62.77 ± 5.28	61.5–81.8	54.3–68.4	10.2	**0.009**	–
BMI (${\mathrm{kg}/m}^{2}$)	21.78 ± 1.60	21.09 ± 0.88	18.5–25.2	19.6–22.5	3.27	0.232	–
Density [D] (${g/\mathrm{cm}}^{3}$)	1.05 ± 0.01	1.06 ± 0.01	1.04–1.07	1.05–1.07	0.95	**0.007**	–
FM (%)	17.56 ± 2.16	14.4 ± 2.00	12.76–21.72	10.81–18.01	21.9	**0.001**	–
FM (kg)	12.19 ± 2.15	8.99 ± 1.08	7.97–15.64	7.03–10.09	26.25	0.100	–
LBM (%)	82.44 ± 2.16	85.6 ± 2.00	78.3–87.2	82.0–89.2	3.9	**0.001**	–
LBM (kg	56.98 ± 4.70	53.78 ± 4.94	50.75–66.68	45.91–58.43	5.61	0.100	–
Roher’s index [RI]	1.22 ± 0.1	1.22 ± 0.1	1.0–1.4	1.2–1.4	0.07	0.982	–
Endomorphy [Endo]	1.58 ± 0.35	1.37 ± 0.16	0.9–2.4	1.2–1.6	15.3	0.09	–
Mesomorphy [Meso]	5.02 ± 1.09	4.99 ± 1.09	2.9–7	3.8–6.6	0.6	0.956	–
Ektomorphy [Ekto]	3.25 ± 0.94	3.2 ± 0.6	1.6–5.4	2.2–3.8	1.56	0.873	–
Training (years)	12.65 ± 4.35	12.75 ± 4.03	4.5–24	8–20	0.79	0.314	–
Age (years)	29.03 ± 4.55	25.00 ± 4.18	19–36	19–33	16.12	0.118	–

Comparative analyses were performed using reference data from the general adult population, as reported by Kalka et al. (27), Żarów et al. (28), and Piechaczek (29). Data from three separate studies were combined due to the absence of a single dataset encompassing all relevant variables.

The study complied with the local Research Ethics Committee recommendations and the Declaration of Helsinki (30). Statistical analysis included the Shapiro-Wilk test (α=0.05) to assess normality. For normally distributed variables (p>0.05), an independent t-test was used; for non-normal distributions (p<0.05), the Mann-Whitney U test was applied. The LASSO method (31) was used for variable selection and regularization, with cross-validation (LASSOCV) to determine the optimal parameter. Python’s *LassoCV* and *SelectFromModel* functions from Scikit-Learn were used to retain the most important variables (see Supplementary Material). Spearman correlation coefficients were calculated for each group to examine the relationships between selected significant variables (see Table 3).

**Table 3:** Statistically significant correlations between the six most important variables identified for the international and national level groups.

Compared variables	Correlation	Significance
International level		
Thigh girth—Body weight	0.722	*
Body weight—Body height	0.912	***
National level		
Thigh girth—Body weight	0.634	***
Thigh girth—Calf girth	0.616	**
Arm relaxed—Body weight	0.596	**
Arm relaxed—Calf girth	0.732	***
Body weight—Body height	0.591	**
Body weight—Calf girth	0.682	***

Asterisks denote significance levels: 0.05 (*), 0.01 (**), 0.001 (***).

## 3 Results

Statistically significant differences between World Cup athletes and top national-level athletes were observed in 23 parameters (see Table 2). The largest percentage differences were found in variables related to body composition. The greatest difference was recorded in abdominal skinfold thickness, which was 30.4% lower (p=0.001) in the international climbers. Other skinfold measurements and body fat percentage also showed significant differences, with lower values in the international group, except for the calf and thigh skinfolds.

The percentage of LBM was 3.9% higher in the international group (p=0.001), as was body density, which was 0.95% greater (p=0.007). Significant differences were also found in body girths, which were mostly smaller among international climbers. The largest differences were noted in the lower limbs: thigh 8.34% (p=0.001), calf 6.3% (p=0.001), and inhaled chest girth 7.82% (p=0.003).

In length-related variables and their respective indices, the greatest difference was observed in body height, which was 3.38% lower (p=0.019) in the international group, as well as in higher values of all upper limb indices in this group. No significant differences in somatotype were found between the groups (see Table 2, Figures 5, 6).

**Figure 1:**
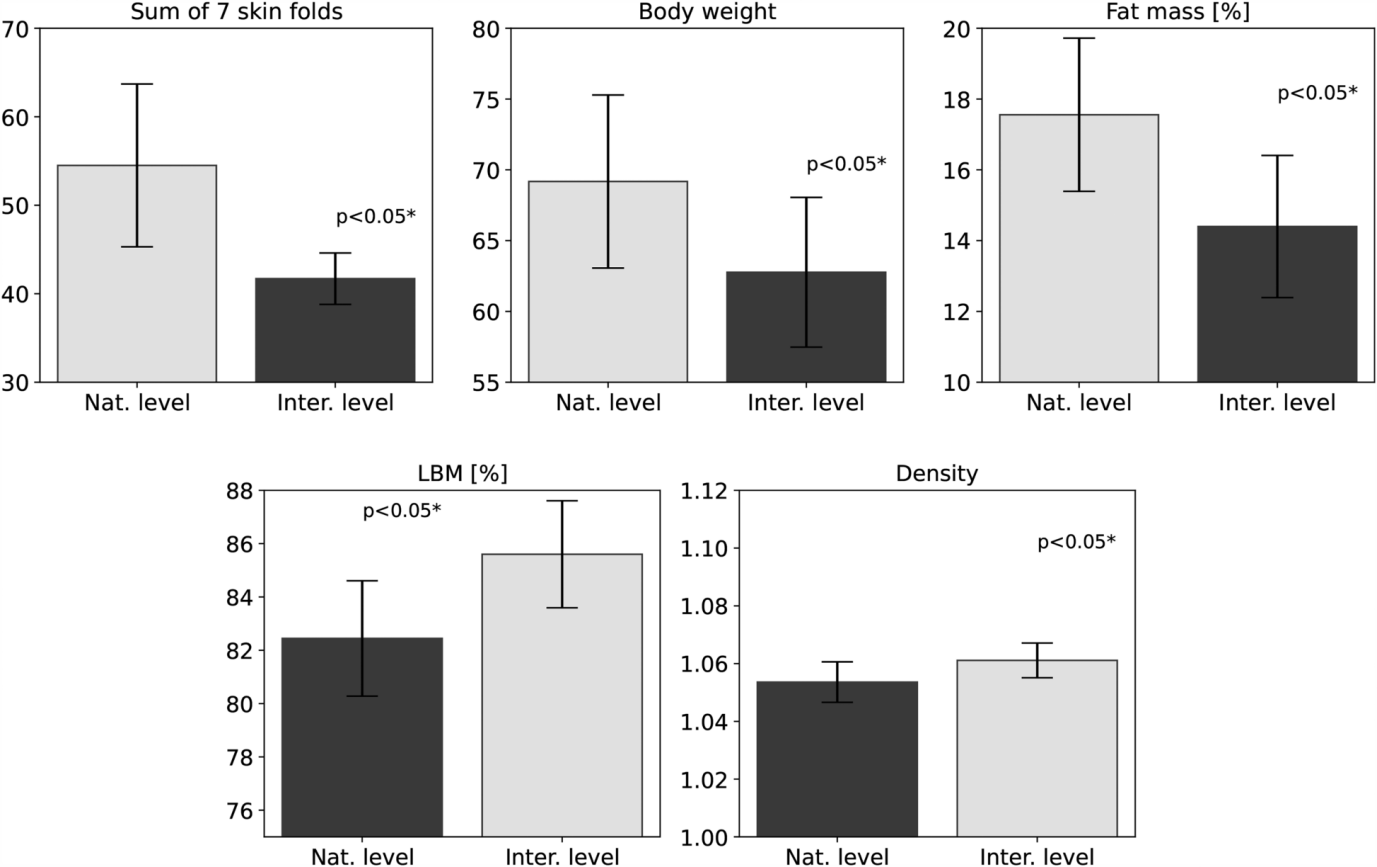
Differences in weight and body composition variables between international and national athletes. Bar charts are shown with standard deviations of the mean.

**Figure 2:**
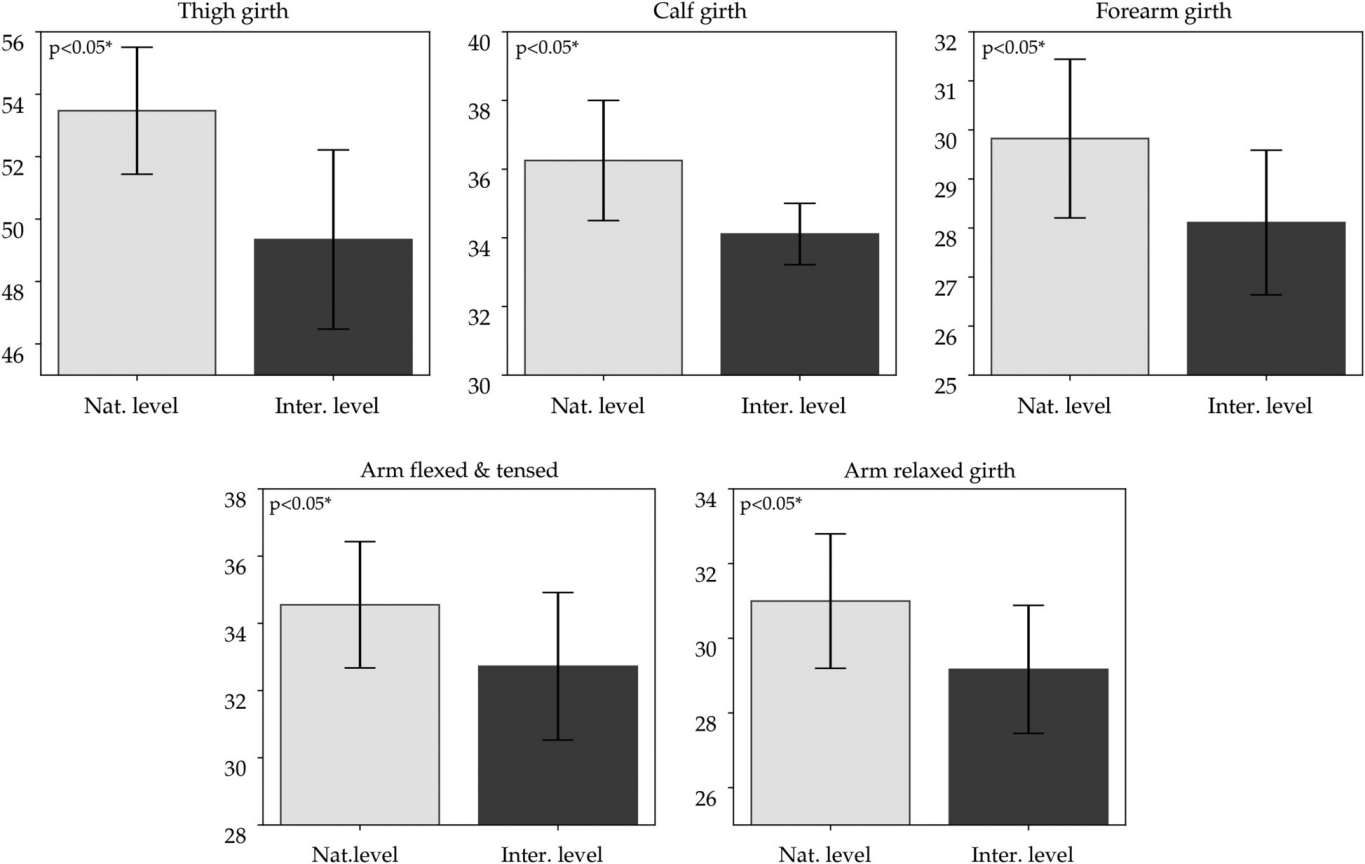
Differences in limb girths between international and national athletes. The bar charts are presented with the standard deviations of the means.

**Figure 3:**
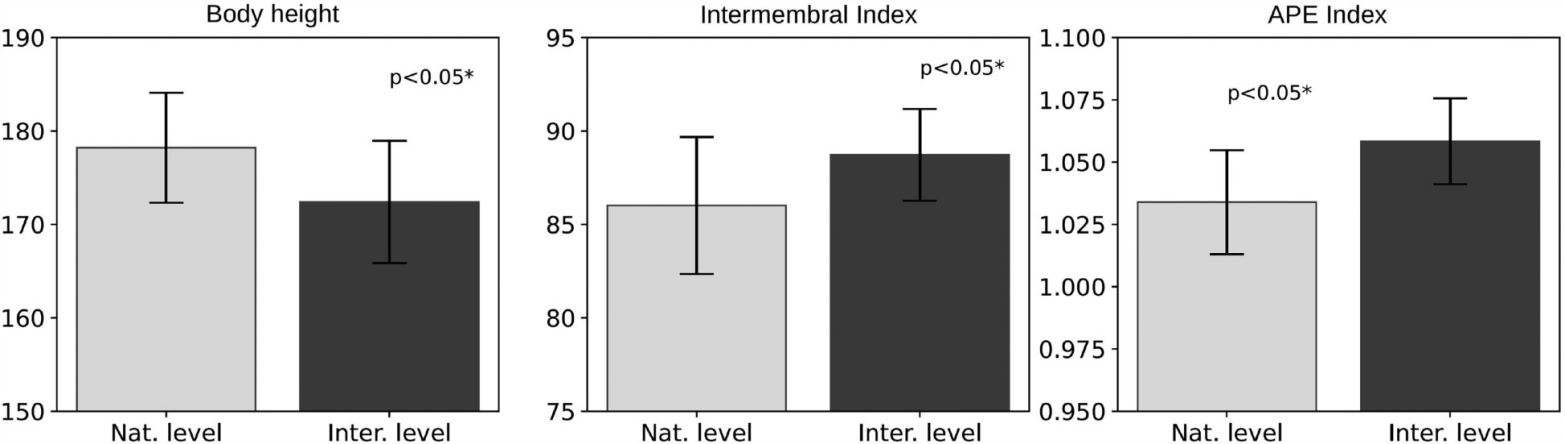
Differences in body length and length indices variables between international and national athletes. Bar charts are shown with standard deviations of the mean.

The most significant parameters are presented in bar charts (Figures 1–3), grouped into three categories representing variables with similar component interpretation. The charts also include standard deviations of the means to illustrate within-group variability.

**Figure 4:**
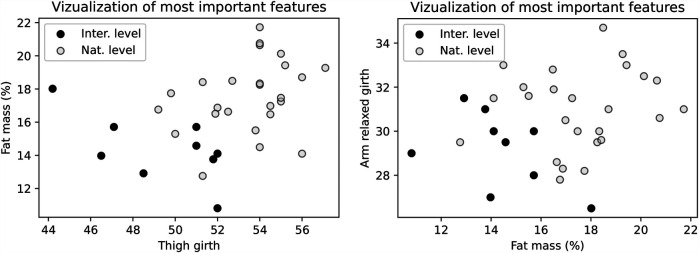
The scatter plots show the three most important variables identified by the optimal feature selection method. The left panel displays the relationship between fat mass and thigh girth, while the right panel illustrates the relationship between fat mass and relaxed arm girth.

Among the 23 statistically significant parameters (Table 2), the LASSO model identified % fat mass, thigh girth, and relaxed arm girth as the three most discriminative variables used for visualization. These were selected from a broader set of relevant features determined by LASSO regression (see Supplementary Material). The scatter plots (Figure 4) illustrate the distribution between the groups of climbers, indicating lower % fat mass and smaller limb girths in the international climbers group.

Spearman’s rank correlation coefficient analysis identified differences in the relationships between anthropometric variables in the international- and national-level groups. In the international-level group (see Table 3), the strongest and most statistically significant correlation was found between body weight and height ($r=0.912^{**},\;p<0.001$). A significant positive correlation was also observed between thigh girth and body weight (r=0.722,p<0.05). In the national-level group (see Table 3), the strongest and most statistically significant correlation was found between calf girth and arm girth ($r=0.732^{*},\;p<0.001$). Other significant correlations included those between body weight and height (r=0.591,p<0.01) and between calf girth and body weight (r=0.682,p<0.01). Additionally, a significant positive correlation was observed between thigh girth and calf girth (r=0.616,p<0.01).

## 4 Discussion

The somatic structure and its impact on performance in sport climbing were examined in international bouldering athletes as compared to national-level climbers and the general adult population. The first notable finding was that the greatest differences between the climbing groups were observed in tissue composition. It is important to emphasize that these differences consisted not only of a lower percentage of body fat among the international climbers, but also a higher percentage of lean body mass in this group. A second notable finding was the observed differences in the limb and chest girths among climbers, particularly in the lower limbs. These differences suggest that climbers at higher performance levels tend to have smaller limb girths. Within the sample, larger limb girths were correlated with higher body weight. This increased weight, in turn, negatively affects climbing performance (37). The third finding was that lower body height combined with proportionally longer upper limbs, as expressed by the Ape Index, Arm Index, and Intermembral Index, was more favorable in international climbers, suggesting that these variables may be important for climbing performance.

**Figure 5:**
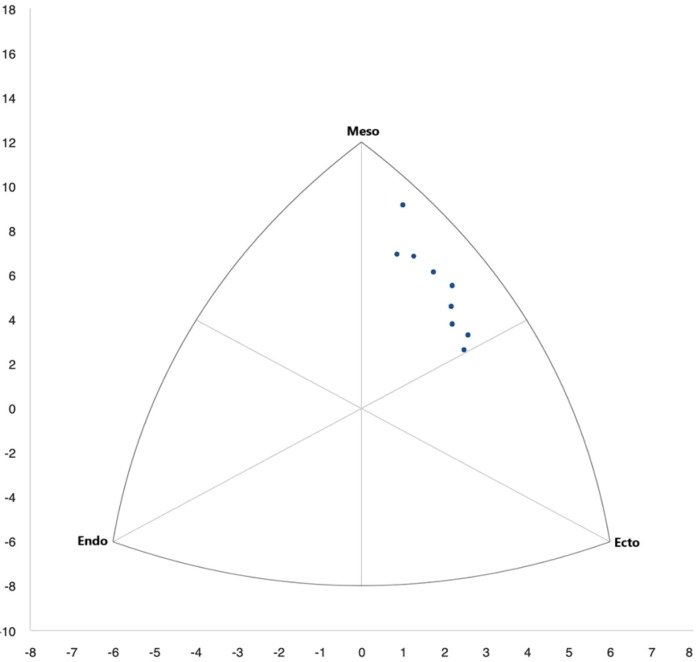
Somatotypes of international athletes marked in blue.

**Body fat assessment** variables revealed the most significant differences in body composition between the athlete groups. The sum of the skinfold thicknesses measured at the triceps, abdomen, and subscapular area was 23.5% lower in the international climber group compared to the national group (see Table 2, Figures 1, 5), suggesting that higher-level climbers tend to have considerably less body fat. Moreover, the individual skinfold measurements (triceps, abdominal, and subscapular folds) also showed significant differences. The international climbers had a 27.6% thinner triceps fold, a 30.4% thinner abdominal fold, and a 28.9% thinner subscapular fold than their national counterparts (see Table 2). However, it is a well-balanced tissue composition, specifically an optimal mix of fat and lean body mass, that is necessary for optimal climbing performance. This may be due to several factors not included in these studies, such as nutrition and training volume (38).

The comparison of these findings with existing literature is somewhat complicated due to the diverse methodologies used across studies. The number and selection of skinfold sites varied considerably and were often not clearly defined (10, 39–41). Errors in locating anatomical measurement points and inconsistencies in protocols, as reported by Pastuszak et al., may further affect the reliability of somatotype and tissue composition assessments (42). Despite these methodological discrepancies, previous studies consistently indicate significant differences in skinfold thickness between climbers and non-athlete adults, as well as between climbers of varying skill levels (43).

Fat mass analysis further confirmed significant differences in body composition between international and national-level climbers. The average fat mass percentage for international climbers was (14.4 ± 2.00%), which was lower than that of national athletes (17.56 ± 2.16%) (see Table 2). This may suggest that at lower levels of sports performance, body fat percentage might be less significant, as it could potentially be compensated by other factors not accounted for in this study. The recorded results are slightly higher than those reported by Macdonald et al. (12) (12.1 ± 4.3%) and Gibson et al. (40) (12.0 ± 3.8%) in studies on national-level bouldering athletes. It is important to note that the higher values observed in this study are accompanied by a smaller standard deviation, indicating potentially more consistent measurements within the sample.

Research consistently indicates that low levels of body fat are a significant factor in enhancing climbing performance (44). Nevertheless, it is important to emphasize that success in this sport depends on the interaction of multiple factors, such as fitness, training volume, and nutrition, all of which influence body composition (45). Low body fat alone does not necessarily ensure athletic success and may, in fact, negatively affect both performance and athlete health (46). In climbing populations, however, body fat percentage is consistently lower than that observed in the general adult population, with mean values of 18.4 ± 2.9% reported by Kalka et al. (27).

**Lean body mass and body density** are commonly used to assess body composition and are linked to athletic performance, particularly in strength-based disciplines such as Olympic weightlifting (47, 48). LBM has also been investigated in sport climbing (49), although some studies omit it entirely (50, 51). In the present study, national-level climbers had higher absolute LBM (56.98±4.60kg) than international-level climbers (53.78±4.94kg), while relative values favored the international group, who showed significantly higher LBM percentages (85.6±2.00%) compared to national-level athletes (82.44±2.16%) (see Table 2). To enable comparison with previous studies, LBM values were expressed as percentages of total body mass. Reported values in the literature include 82.35% observed by Macdonald et al. (12), which are generally higher than those found in the general adult population, such as 62.59% reported by Żarów et al. (28). These findings suggest that relative LBM helps to distinguish climbers from non-athletes, although it remains unclear whether it can differentiate between performance levels. Increased LBM may also play a role in injury prevention. Research by Grønhaug et al. (52) shows that elite climbers experience fewer injuries than intermediate climbers. Increased strength may protect joints and ligaments and has been shown to reduce injury prevalence (53). Therefore, the findings of previous studies may be partially explained by the higher muscle mass observed in elite climbers, who typically have more muscle mass than control groups.

Body density, defined as the ratio of body mass to volume, is another parameter used to estimate body composition (54). Although research in climbers is limited—primarily involving bouldering specialists (22) and some elite lead climbers—available data indicate higher values in international-level climbers $\left(1.06\pm 0.01\;{g/\mathrm{cm}}^{3}\right)$ compared to national-level athletes $\left(1.05\pm 0.01\;{g/\mathrm{cm}}^{3}\right)$ (see Table 2). These findings align with those of Ozimek et al. (22), who reported similar values $\left(1.06\pm 0.008\;{g/\mathrm{cm}}^{3}\right)$, and exceed population norms, such as $\left(1.05\pm 0.007\;{g/\mathrm{cm}}^{3}\right)$ reported by Piechaczek (29). These results suggest that bouldering athletes demonstrate higher body density than the general adult population, and that differences in this parameter may also exist between climbing disciplines. However, these assumptions require further verification through broader studies.

**Body weight** is a significant factor in climbing performance and has been emphasized by researchers as critical to success in the sport (55). In the present study, significant differences were observed in body weight when comparing the general adult population and recreational climbers to more advanced athlete groups. The average body weight of the general adult population (27) was higher, at (77.11 ±9.3 kg), compared to the national (69.18 ±6.11 kg) and international (62.77 ±5.28 kg) athletes. The values obtained in the present study were significantly lower in athletes at the international level compared to national-level athletes, as reported by Riley et al. (56) (69.5 ±9.8 kg). These findings are consistent with previous research indicating that lower body mass is associated with greater relative strength, an important determinant of climbing performance (57). The negative correlation between an athlete’s body mass and their performance in climbing-specific tasks may partly explain the greater performance observed among climbers specializing in bouldering, as demonstrated by Buraas et al. (58).

**Morphological indices** based on body mass and stature, such as BMI and Rohrer’s Index, showed no significant differences between climbers of different performance levels. In the present study, BMI was (21.09±0.88) for international-level climbers and (21.78±1.60) for national-level athletes, aligning with previous findings (59) (see Table 2). Compared to the general population (23.1±2.9) (27), climbers showed lower BMI values, similar to those reported by Medernach et al. for recreational climbers (22.3±1.5) (60). The relationship between BMI and climbing performance remains unclear, with studies reporting positive (40, 61), negative, or no associations (41, 62). Grønhaug (63) found no correlation between BMI and climbing performance or chronic injuries, noting similar values in recreational and elite climbers and questioning the need for extremely low BMI values in this population. In contrast, studies in sports involving contact and rapid direction changes associate higher BMI with injury risk (64–66). Thus, such findings are not directly applicable to climbing. Campa et al. (67) suggest that BMI is not a reliable measure of body composition in athletes and should be complemented by more specific assessments. According to Wanke’s somatotype classification (68), Rohrer’s Index values below 1.24 indicate a slim body type. In this study, the average Rohrer’s Index was 1.22 in both groups (see Table 2), supporting the slim body type typical for climbers, consistent with the value of 1.23 reported by Ozimek et al. (19). For comparison, the general population average of 1.25 (29) underscores the leaner build observed in climbers.

**Body height** is an important parameter that differentiates climbers at various performance levels. In this study, World Cup athletes were on average 3.38% shorter than national-level climbers (see Table 2). This trend is consistent with previous studies, which also reported lower average height among high-level bouldering athletes compared to lower-level competitors (12, 40, 69). In contrast, the general adult population has a higher mean body height of (180.9±7.2cm) (27). Although shorter stature may benefit bouldering, studies report inconsistent associations between height and performance (70). These aspects should be considered in future research, particularly in relation to performance level and bouldering setting trends (71).

**Upper limb proportions** were found to significantly influence climbing performance. In this study, international climbers had a higher Ape Index (1.06±0.02) compared to national-level athletes (1.03±0.02), supporting its potential relevance despite conflicting findings in previous research (72–74). The Intermembral Index, defined as the ratio of upper to lower limb length, was also significantly higher in the international group (88.72±2.46) vs. (86.01±3.66), which is consistent with earlier results (22). The Arm-Index, calculated as upper limb length relative to body height and excluding shoulder width, was higher among international climbers (46.22±1.26) than among national climbers (44.93±2.07). This index reflects vertical reach, which may be more relevant in climbing. The current findings align with previous studies on both bouldering (22) and lead climbing (75), although direct comparisons are limited due to data normalization in those publications. Larger and more diverse samples are needed to verify the Arm-Index as a climbing-specific anthropometric measure.

**Girths** measurements in the present study indicated that smaller circumferences of both upper and lower limbs, as well as reduced chest circumference during inhalation and exhalation, are characteristics of international-level climbers (see Figures 2, 4). A similar observation was reported by (39), although they noted this pattern only for the lower limbs. This pattern suggests that climbers may optimize the development of limb musculature, avoiding unnecessary increases in overall body mass (76).

**Figure 6:**
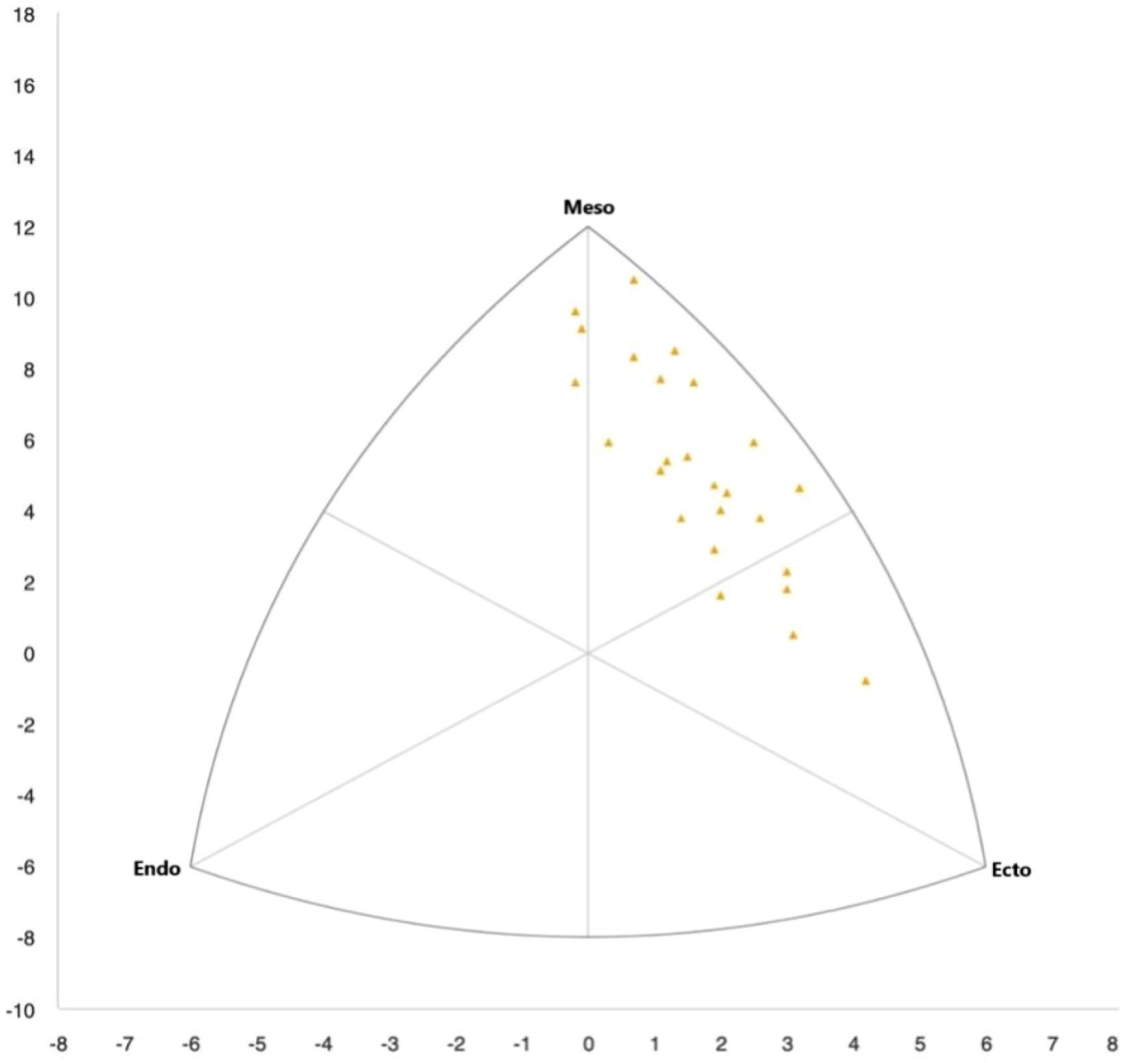
Somatotypes of national athletes marked in yellow.

**The somatotype** has an important role to play in identifying the physique characteristics best suited to the demands of a given sport. It can support talent identification and help coaches tailor training to maximise performance (77). However, comparing somatotypes across competition levels is difficult, as differences may result not only from sport-specific requirements but also from ethnic and racial characteristics (78). In the present study, no statistically significant differences in somatotype were found between international and national climbers. The most noticeable variation appeared in endomorphy, reflecting greater fat accumulation among national athletes (see Table 2, Figures 5, 6), though not statistically significant. According to the Heath-Carter method (23), athletes were classified as ectomorphic mesomorphs, with mesomorphy dominant. In a study by Ozimek et al. (22), climbers showed a different profile: higher endomorphy (1.92±0.53), lower mesomorphy (3.70±0.98), and ectomorphy (3.16±1.05), indicating a leaner, less muscular build. As existing results remain inconsistent, further research with larger and more diverse samples is needed to clarify the role of somatotype in climbing performance.

## 5 Conclusions

The results of the present study suggest that international elite climbers had lower body fat and higher lean body mass when compared with national level climbers and general adult population. Still, body fat did not significantly differentiate national level climbers from general adult population. The findings of this study suggest that low body fat comes from higher training volumes rather than calorie restriction. Differences were also observed in limb and chest circumferences, especially in the lower limbs, where smaller girths correlated with lower body weight - an important factor affecting performance in sport climbing. This study confirms that upper limb proportions significantly affect climbing performance. International climbers showed higher values in Ape Index, Intermembral Index, and Arm Index compared to climbers at the national level, with statistically significant differences. Future research should focus on the long-term effects of climbing on the body composition of both men and women in all climbing disciplines, using standardized research methods.

## 6 Practical applications

Climbing training should be focused on developing strength and power without excessive muscle hypertrophy, which leads to increased body mass and may negatively impact sports performance, while maintaining an appropriate balance between fat and muscle tissue. Understanding the differences between climbers and the general adult population can support recreational or novice climbers in achieving optimal anthropometric characteristics through targeted training. Careful assessment of body composition and its optimization by appropriately trained personnel can support the training process and prevent the possible disorders associated with excessive weight loss which occur in climbers.

## 7 Strengths and limitations

The study presented here has several limitations that need to be taken into account. The small sample size of international and national climbers limits the generalizability of the results to a wider population of climbers. In addition, the groups were not evenly distributed, with different sample sizes, which complicates statistical analysis and may affect comparisons between international and national climbers. The study also did not distinguish between training adaptations and genetic predispositions, and it focused only on male climbers—which limits its applicability to female athletes. Furthermore, the measurement methods used, although widely accepted for collecting field data, have inherent limitations. They are less precise than more advanced techniques such as DEXA scans, which could not be used due to logistical constraints. An additional consideration is that the estimation of body fat based on BMI in the international group may have been influenced by the ethnic composition of the sample, as a substantial proportion of these athletes were of Asian origin, who, according to Sung et al. (79), tend to exhibit higher body fat percentages at relatively lower BMI values compared to Europeans. Differences in competition schedules may also have affected the results, as international athletes may have been in a different state of nutrition and training than national athletes at the time of measurement. Furthermore, it remains unclear whether body composition was influenced by factors such as water retention or dehydration related to competition participation. Despite these limitations, the study has notable strengths. It provides rare and valuable data on the somatic constitution of top international climbers. Given the scarcity of such comprehensive datasets, this study contributes significantly to the understanding of anthropometric factors related to performance. Additionally, the inclusion of both international and national climbers allows for a comparative perspective, further enriching the analysis.

## Data Availability

The raw data supporting the conclusions of this article will be made available by the authors, without undue reservation.
