# Supplementary material for: Morphology of male world cup and elite bouldering athletes
Source: Front Sports Act Living. 2025 Jun 11;7:1588414. doi: 10.3389/fspor.2025.1588414 (PMC12198683; doi:10.3389/fspor.2025.1588414)
Supplement: Supplementary file 1 [file Datasheet1.pdf]

The Supplementary Material described the variable selection procedure based on *LassoCV* and *SelectFromModel*, consisting of the following steps:

1. Training a Lasso regression model with *LassoCV*: the method was applied to the training data to fit the model and assign significance scores to each variable.

2. Assessing variable relevance: the variables were evaluated based on their contribution to predictive accuracy.

3. Selecting significant variables: *SelectFromModel* used the scores provided by *LassoCV* to identify and retain the most relevant ones.

4. For visualisation, three variables—thigh girth, relaxed arm girth, and fat mass—were selected. Their combinations (fat mass % vs. thigh girth, left; fat mass % vs. relaxed arm girth, right; see fig. 4) clearly separated the compared groups.
